# Advanced Echocardiographic Imaging of the Congenitally Malformed Heart

**DOI:** 10.2174/1573403X11309030008

**Published:** 2013-08

**Authors:** D Black, J Vettukattil

**Affiliations:** Wessex Cardiothoracic unit, Congenital Cardiac Centre, Southampton University Hospital NHS Trust Tremona Road Southampton UK, SO166YD, UK

**Keywords:** Three dimensional echocardiography, Deformational imaging, TMAD.

## Abstract

There have been significant advancements in the ability of echocardiography to provide both morphological and functional information in children with congenitally malformed hearts. This progress has come through the development of improved technology such as matrix array probes and software which allows for the off line analysis of images to a high standard. This article focuses on these developments and discusses some newer concepts in advanced echocardiography such is multi-planar reformatting [MPR] and tissue motion annular displacement [TMAD].

Our aim is to discuss important aspects related to the quality and reproducibility of data, to review the most recent published data regarding advanced echocardiography in the malformed heart and to guide the reader to appropriate text for overcoming the technical challenges of using these methods. Many of the technical aspects of image acquisition and post processing have been discussed in recent reviews by the authors and we would urge readers to study these texts to gain a greater understanding [1]. The quality of the two dimensional image is paramount in both strain analysis and three dimensional echocardiography. An awareness of how to improve image quality is vital to acquiring accurate and usable data.

Three dimensional echocardiography (3DE) is an attempt to visualise the dynamic morphology of the heart. Although published media is the basis for theoretical knowledge of how to practically acquire images, electronic media [eg.www.3dechocardiography.com] is the only way of visualising the advantages of this technology in real time.

It is important to be aware of the limitations of this technology and that much of the data gleaned from using these methods is at a research stage and not yet in regular clinical practice.

## THREE DIMENSIONAL ECHOCARDIOGRAPHY

### Functional and Volumetric Assessment

#### Right Ventricle

The use of 3DE to assess the right ventricle is an area of interest. Assessment of right ventricular volumes plays an important role in the management of patients with congenital heart disease. 3DE has been shown to be reproducible and more reliable than 2DE in assessing right ventricular volumes when correlated with CMRI in patients with Tetralogy of Fallot and normal controls [2]. Real time 3DE (RT3DE) assessment of right ventricular volumes using intra operative TOE has been performed and was felt to improve the quantitative evaluation of the RV during surgery [3]. Van der Swaan *et al*. demonstrated 95% sensitivity and 89% specificity for RT 3DE in identifying RV dysfunction in patients with congenital heart disease [4]. Although these studies show significant promise for the assessment of the right ventricle using 3DE, one needs to stress the importance of obtaining good images for assessment. The off line analysis is still challenging and time consuming and we feel is open to significant intra-observer variability [5]. Another current limitation is the inability to capture the full RV volume within the sector particularly in dilated and abnormal RV, where assessment of volumes is important.

#### Left Ventricle

Left ventricular volumes, mass and ejection fraction have been assessed in neonates and infant with congenital heart disease [6]. The study by Friedberg *et al.* demonstrated that 3D measurements of mass and volume correlated well with MRI. 3D derived EF was smaller than MRI by 9.3%(p<0.001). The echocardiogram in this study were performed directly after the MRI scan, the general anaesthesia received for MRI would certainly have made performing the echocardiogram in these children practically easier. A concern in this age group is the amount of movement artefact which affects image quality and in our experience makes analysis less reliable. In older children LV volumes determined by 3DE have also been show to correlate with MRI derived EF [7]. Once again 3D echocardiography tends to underestimate MRI derived volumes. The interesting point made in this paper relates to the importance of quantification software settings and the difference these can make to the data. An awareness of the different analysis software available is important to ensure the reproducibility of the data. Hascoet *et al.* have compared QLAB 6.0[Phillips] which is a semi-automated system with TomTec 4D LV which is primary manual tracking with semi-automated border detection. They found that 3D volume assessment was feasible using both systems with good correlation of LV volume measurements [8]. When performing 3D volumetric assessment, particularly with semi automated border detection, stitching artefacts will impair the ability of the software to track the image. It is important to record the image over a number of loops and ensure minimal movement during acquisition. Ongoing advances in the ability for single loop acquisition of images will improve quality.

### Morphological Assessment

The heart is a moving three dimensional structure and in congenital heart disease can be extremely complex. The use of 3DE in the assessment of congenital heart disease makes sense. 

#### Assessment of Morphology.

RT3DE has been shown to be superior to 2DE and 2D TOE in assessing atrioventricular valves in patients with congenital heart disease [9]. In this study surgical findings were used as the reference standard and RT3DE provided superior detail of the mural leaflet and anterior commissural abnormalities for the left AV valve. 3DE with MPR has also been used in the assessment of patients with Ebsteins initially diagnosed using 2DE, using 3DE 48% of the patients were re classified as having tricuspid valve dysplasia due to new information obtained [10]. A study by the same group revealed new information about the mechanism of action of the mitral valve. Bharucha *et al*. showed mitral valvar annular area in children decreases in diastole, and increases in systole. In those with mitral regurgitation, the annulus is dilated and the dynamic annular function is depressed [11]. In our institution 3DE has been show to impact significantly on preoperative decision making in a number of congenital heart defects [12]. New, clinically important information, which altered management or changed the principal diagnosis, was obtained in 32 (11%) cases. This determined suitability for biventricular repair in 11 patients, clarified the morphology of atrioventricular valves in 7, helped in assessment of aortic, mitral, or prosthetic valvar disease in 13, and identified a vascular ring in the other patient.

3DE has also been shown to be useful in demonstrating the deep trabeculations associated with left ventricular non compaction and has provided insights into the morphology of sub aortic stenosis [13, 14].

The use of 3D echocardiography to define cardiac morphology is becoming increasingly frequent and more accurate. A recent case report describes the identification of a supramitral ring in a 2 month old which allowed for full assessment of the fibrous shelf prior to surgery [15].

3DE and RT3DE have a significant role to play in the assessment of morphology in congenital heart disease. With improved operator skills and ease of data analysis, including the ability for bed side analysis this role will continue to become more clinically significant. 

Multi-planar reformatting is a vital technique for dissecting and understanding of a congenitally malformed heart. For a full description of the technique please see the following reference [1].

#### Antenatal Diagnosis

Many published studies have shown that application of three-dimensional and real-time 3D ultrasound modalities can improve certain aspects of fetal echocardiography, but have left open the question of whether these modalities improved the accuracy of prenatal detection of anatomical fetal cardiovascular malformations [16]. In a study by Yagel *et al.* 3DE added value in 6% of cases of fetal anatomical cardiovascular anomalies. Notably was the diagnosis of two foetuses with agenesis of ductus venosus to the coronary sinus and two with total anomalous pulmonary venous connection diagnosed with MPR. They do note that the overall contribution was minimal, but with improving technology and resolution 3DE could make a more significant impact in antenatal diagnosis.

#### Catheter Laboratory

As previously mentioned 3DE has played an increasing role in the pre catheter management of patients with congenital heart disease. This is improving clinician awareness of what the procedure may involve and guide the management decisions.3DE has a vital role to play in guiding transcatheter procedures for both septal and valvar structures [17].

Scheurer *et al*. demonstrated the use of live 3DE to guide the performance of endomyocardial biopsy in children [18].

#### Atrial Septal Defects and Patent Foramen Ovale

**Fig. (1):**
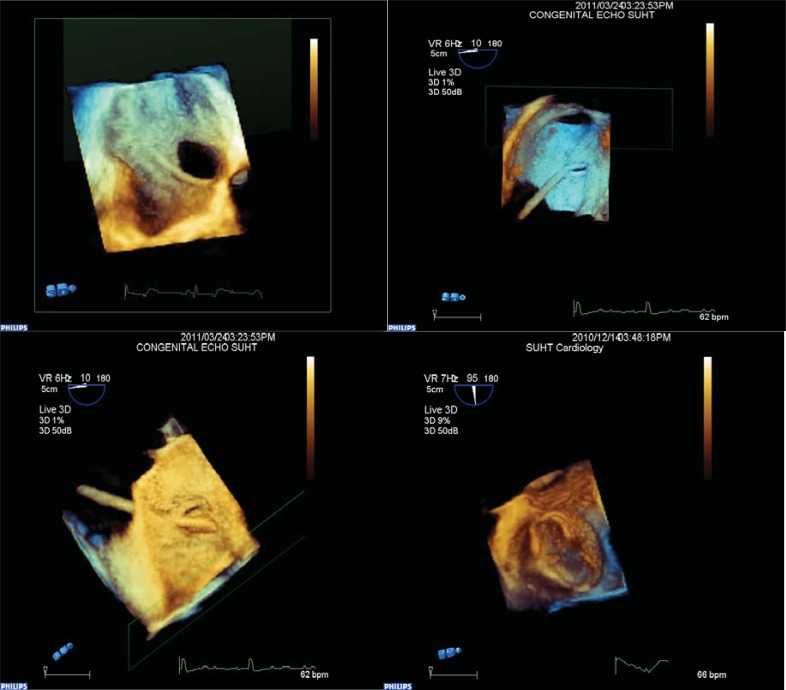
Images captured during real time three dimensional transoesophageal echocardiography for device closure of patent foramen ovale and atrial septal defects. Image in top corner left demonstrates the anatomy of an atrial septal defect from right atrial aspect with inferior vena cava entering inferiorly. Next image to the right demonstrates catheter passing through a patent foramen ovale from right atrial aspect and the following image demonstrates the catheter entering from the left atrial aspect in the same patient. Final image demonstrating device in cross section from left atrial aspect.

The use of 3D TOE to guide catheter intervention in congenital heart disease is well established [19]. In our cardiac catheter laboratory we routinely use RT3D Trans-oesophageal echocardiography (TOE) for the device closure of patent foramen ovale and atrial septal defects. These images have provided valuable insight into the morphology of the atrial septum and have influenced management. The use of RT3D TOE in this context has reduced the need for fluoroscopy and shortened the procedure time. We are also able to avoid balloon sizing the defect and thus improve procedure time and avoid possible procedure related injuries (Fig. **1**).

#### Ventricular Septal Defects

Transcatheter closure of ventricular septal defects is more challenging and carries the additional risk of complete heart block. Accurate morphological assessment and location of the VSD are crucial prior to the procedure. 3DE provides accurate information on the location and size and morphology of the septal defect. 3DE has been used to guide transcatheter closure of VSD particularly in the context of a complicated post infarct VSD [20].

#### Electrophysiology and Trans-septal Puncture

RT 3D TOE allows high-quality 3D visualization of the atria, pulmonary veins, and the mitral valve annulus less invasively during electrophysiological procedures. The addition of the third dimension is also useful during trans-septal puncture in patient’s under-going left atrial procedures such as flutter ablation [21, 22].

#### Mitral and Aortic Valve Disease

Advances in percutaneous methods for repair of mitral and aortic valves are applicable to patients with congenitally malformed hearts. Recently techniques have been developed allowing percutaneous repair of both the aortic and mitral valve [23, 24].

The use of RT 3D TEE for the guidance of mitral valve interventions allows for adequate visualization of the mitral valve and its surrounding structures for precise placement of devices in the coronary sinus during annuloplasty procedures and during edge-to-edge repair of the mitral valve using MitraClip [25]. The relation of the anterior leaflet of the mitral valve to the LVOT can be depicted with high precision using RT 3D TOE aiding in the accurate placement of these stent based prosthesis [CoreValve] for the treatment of aortic stenosis. Sadagopan *et al.* showed that 3DE provided accurate measurements of aortic valve annulus and number of valve leaflets, in identifying sites of fusion of the leaflets as well as nodules and excrescences that characterized dysplastic valves [26].

**Fig. (2):**
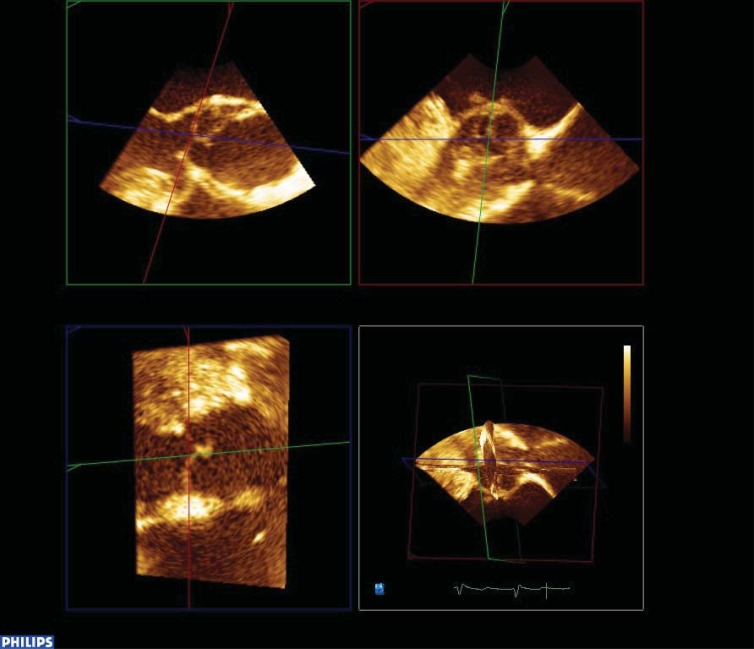
The use of multi planar reformatting in the assessment of a patient with aortic valve stenosis. This image demonstrates the views of the aortic valve obtained from 3 orthogonal planes. The image in the bottom right hand corner is the three dimensional reconstruction of the area of interest.

3DE is now routinely used in the assessment of the aortic valve annulus prior to percutaneous balloon valvuloplasty in our catheter lab. 3DE measurements have been show to correlate more closely to angiographic measurements and we feel that this avoids potential balloon under sizing for the procedure [unpublished data] (Fig. **2**).

#### Left Atrial Appendage Closure

There has been a significant increase in procedures for the percutaneous closure of the left atrial appendage (LAA) following the Percutaenous Left Atrial Appendage Transcatheter Occlusion (PLAATO) trial which demonstrated the feasibility and the safety of this process [27].

The orifice of the left atrial appendage should be measured in two orthogonal views to select the most appropriate device size. RT3D TOE will improve the accuracy of the measurements by providing views where the left atrial appendage orifice could be visualized and measured without geometric assumptions. 

There have been two studies comparing 3D TTE with 2D TOE in identifying LAA thrombus. No LAA thrombus was missed with 3D TTE and it also offered the advantage of better distinguishing pectinate muscles from thrombi [28, 29].

3D Echocardiography continues to develop and will play a more significant role in a number of clinical areas in the future. There are a number of current reviews discussing 3D echocardiography in detail [30].

## CARDIAC DEFORMATION IMAGING USING SPECKLE TRACKING ECHOCARDIOGRAPHY

Strain and strain rate give us measures of cardiac deformation and contractility respectively. Strain and strain rate have also been determined using tissue Doppler but for the purposes of this review we will be focussing on speckle tracking echocardiography.

Congenital heart disease faces a number of challenges when attempting to assess ventricular function due to complex anatomy, variable loading conditions and the fact that the right ventricle with all its complexity is as important as the left ventricle. Speckle tracking echocardiography has the potential to advance the understanding of cardiac function in congenital heart disease overcoming these obstacles.

The introduction of new technology comes with a number of difficulties. Different vendors will have different solutions for the same problem, and in the context of this one needs to ensure the standardisation of acquisition and analysis of data. It is then important to acquire data on feasibility, reliability and accuracy of the method and to establish a set of normal values as a point of reference.

Speckle tracking echocardiography is in the early stages of this process. Although a number of studies have been published on its application in various aspects of congenital heart disease it is not yet used for regular clinical decision making. The limitations of speckle tracking echocardiography have been outlined by a number of experts in this field [31-33]. It is important to have a good practical approach to using this technique for routine clinical practice and to make use of guidance available in the literature [34, 35].

Different vendors produce different results. Koopman *et al.* compared results obtained from strain analysis using Echopac, SPEQLE and QLAB Phillips [36]. They found reasonable agreement between vendors for longitudinal and circumferential strain but not for radial strain. They also looked at vendor independent software Tomtec and found only good agreement for longitudinal strain. Interestingly in this study the inter and intra observer variability for strain measurements was reported as good for longitudinal and circumferential strain, the coefficient of variation was about 10% for this measurement. This is one of the current problems with this technology as it is difficult to make clinical decisions based on a measurement that varies by 10% between observers. Radial strain and strain rate measurements showed even wider variation and were poorly reproducible.

**Fig. (3):**
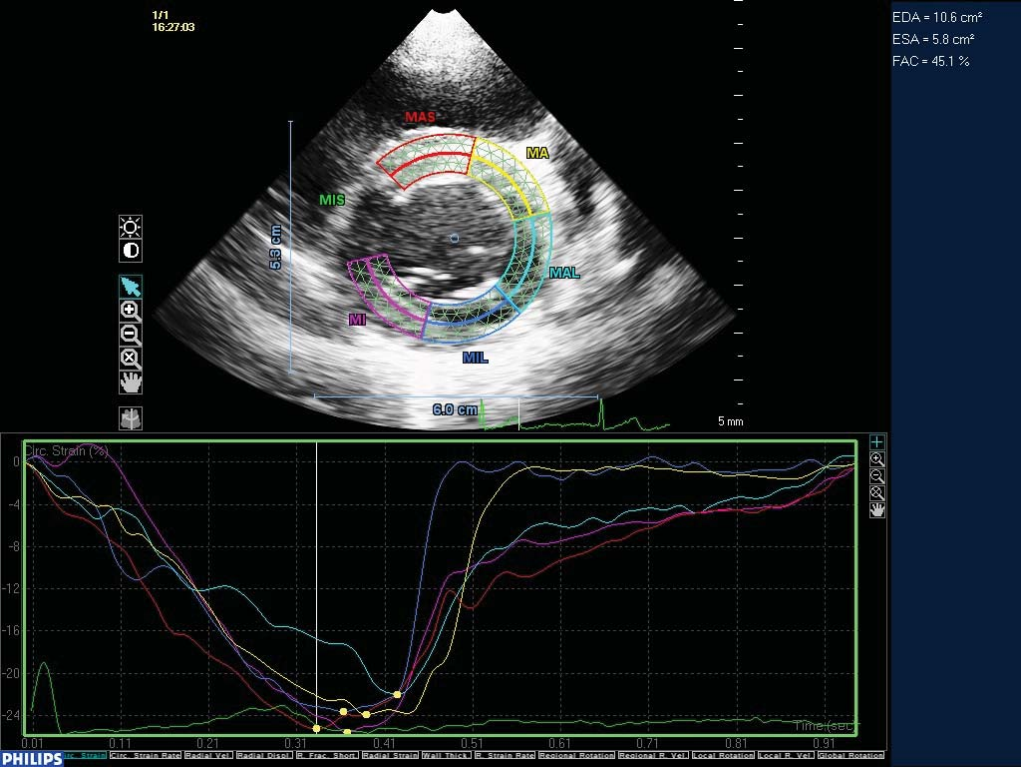
Two dimensional trans thoracic view of base of the left ventricle in short axis. Two dimensional speckle tracking demonstrating deformation in 6 short axis segments. Mid antero-septal [MAS], Mid anterior [MA], Mid antero-lateral [MAL], Mid inferolateral [MIL], Mid inferior [MI] and Mid infero-septal [MIS]. The MIS segment has been excluded automatically due to poor tracking through the cardiac cycle. Graphic display of deformation shows synchronous contraction through all segments in this normal 9 year old volunteer.

Only fairly recently have normal values been published in children [37]. Although this data is a significant further development more normal data is needed. We still have not defined the age related variation in longitudinal and circumferential deformation which is vitally important in understanding strain and in accurately assessing function in patients with congenital heart disease (Fig. **3**). Recent work has been published for effect of age and gender on left ventricular rotation and twist in normal adults which showed an increase in LV rotation and twist with age [38]. This acquisition of normal data is an important ongoing process which will help to incorporate these techniques into routine clinical practice.

The benefits of speckle tracking echocardiography are that it is angle independent, it is a two dimensional measure and can be performed at lower frame rates (preferably more than 60Hz) than tissue Doppler. Strain rate measures determined from speckle tracking echocardiography are generally lower than colour tissue Doppler measures due to the lower frame rates and that they measure mean strain rather than peak strain [36].

With these limitations in mind the following paragraphs discuss some of the recent findings using STE.

### Assessing the Single Ventricle

2DSTE derived global strain has been shown to agree with that derived by tagged CMRI [39]. This study showed reduced global strain in morphological left ventricles following fontan operation for tricuspid atresia. There was also a loss of the normal apicobasal increase in regional deformation that is observed in normal left ventricles. The authors conclude that strains measured by 2DSE agree with strain measured by magnetic resonance imaging globally but vary regionally and that global strain may be a more robust tool for cardiac functional evaluation than regional strain in SV physiology.

Patients post Fontan operation with single LV morphology have been shown to have reduced strain and strain rate when compared with age matched controls [40]. This was demonstrated at a relatively young age and the significance of this needs further studies. Current data from strain analysis in this context is difficult to interpret and is currently not used to guide management decisions. The data may serve as a baseline for an individual patient which can subsequently be used to monitor for any deterioration in function.

A benefit of STE is the ability to assess deformation independent of ventricular geometry. This has been used to assess the deformation properties of morphological right versus left ventricles in patients following Fontan operation [41, 42]. They concluded that deformation and overall ventricular longitudinal deformation was not different between morphologic right and left ventricles. These findings may reflect similar adaptation of longitudinal function of both ventricular morphologies to the single-ventricle circulation.

The changes in a morphological right ventricle following a Norwood operation have recently been studied by Petko *et al*. [43]. They found that two-dimensional global and regional longitudinal strain and strain rate as well as tricuspid annular plane systolic excursion were reduced in patients with hypoplastic left heart syndrome after the Norwood operation. These findings were based on echocardiographic examinations before and 21 days after the procedure and they were unable to explain this decrease based on any of the examined preoperative and postoperative patient or surgical factors. This study demonstrates our current understanding of the significance of this deformation data, certainly more studies are needed to improve our understanding of their significance and how to apply this to clinical practice.

### Lesions Affecting Loading Conditions

In adult patients STE has shown interesting changes in right and left ventricular deformation following percutaneous closure of atrial septal defects [44]. RV global longitudinal strain decreased significantly following closure and LV circumferential strain increased significantly correlating with an increase in LVEDV and LVCO. The authors conclude that longitudinal strain of the right ventricle works as indicator of right ventricular function dependent on loading conditions while SR seems to be less dependent on it. Circumferential strain could be used as an indicator of left ventricular response to normalized loading conditions. 

The dependence of deformation on loading conditions is extremely important to consider should it be used for clinical decision making in patients with congenital heart disease. This effect has also been demonstrated using 2DSTE in patients following percutaneous pulmonary valve implantation [PVI] in the context of combined pressure and volume loading [45]. In this study PVI led to RV unloading with increased strain and strain rate in the RV septal and free walls. These two studies demonstrate the effect of loading conditions on cardiac deformation, a decrease in pre-load with atrial septal defect closure resulted in a reduction in the degree of deformation and a decrease in after-load following PVI resulted in an increase in the amount of deformation. It would be interesting to assess the initial response of the RV to increased after-load, which may be an increase in deformation and subsequently a chronic increase my result in the reduced deformation seen prior to PVI in this study.

**Fig. (4):**
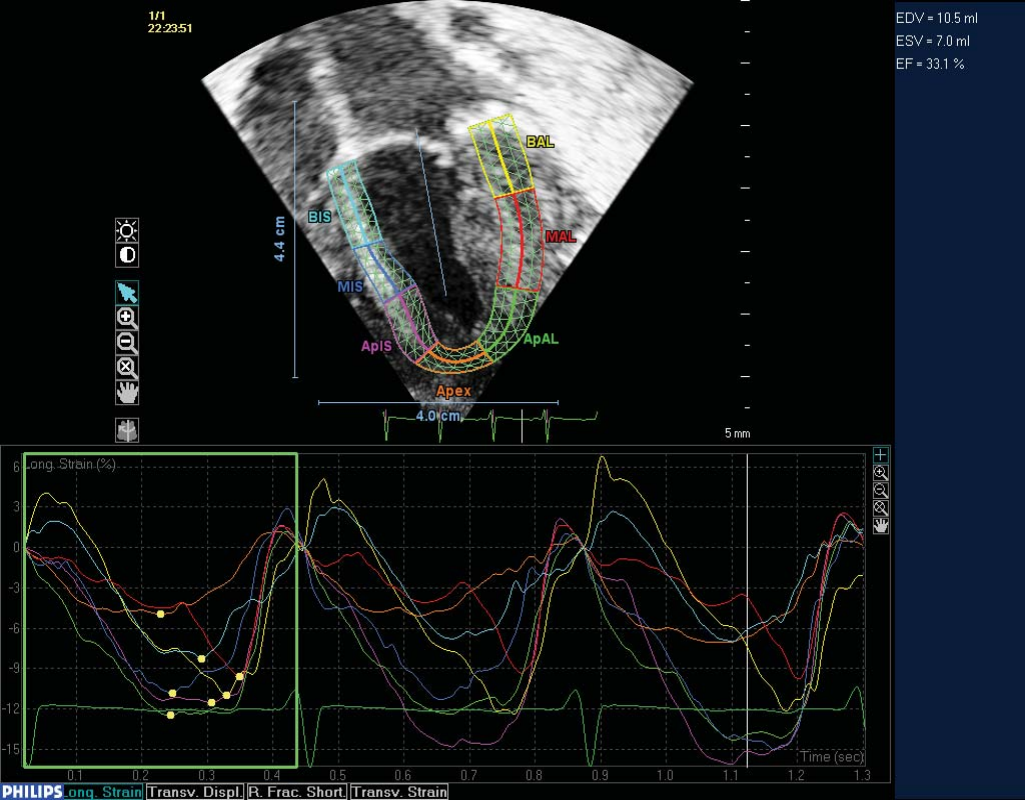
Apical four chamber view of the left ventricle in a neonate post surgical aortic valvotomy. Two dimensional speckle tracking demonstrating longitudinal deformation of the left ventricle. The ventricle is divided into 7 segments: Basal inferior septal [BIS], Mid inferior septal [MIS], Apical inferior septal [APIS], Apex, Apical anterior lateral [ApAL], Mid Anterior Lateral [MAL] and basal anterior lateral [BAL]. A graphic display of deformation of the segments through three cardiac cycles is also demonstrated. This shows a reduction in deformation in this patient in the initial post operative period.

This pathophysiology has also been noted in other conditions such as aortic stenosis where there is an increase in deformation initially in response to increased after load and with chronicity and severity both strain and strain rate become reduced [46, 47] (Fig. **4**).

In patients with TGA deformation in the systemic RV differed significantly from that in the normal RV, presumably due to increased after load [48]. The systemic RV also demonstrated a shift from longitudinal to circumferential deformation. In adults with TGA the systemic right ventricular longitudinal strain is reduced, this has been found to be a predictor of adverse clinical outcome in patients with atrial switch [49].

In patients with obesity and a structurally normal heart it has been shown that both strain and strain rate become reduced over time [50]. Recently evidence suggests that obesity in children has a significant impact on regional myocardial deformation with a reduction in longitudinal strain and strain rate [51]. Unpublished data from our institution shows that children with raised BMI demonstrate increased regional deformation in the basal septal segment of the left ventricle. It is possible that this is an early change in a physiological process reflecting the hearts response to an increased preload generated by increased venous return in individuals with raised BMI.

A recent interesting paper has demonstrated the effect of loading conditions on myocardial deformation in the context of twin to twin transfusion syndrome. This study demonstrated that in twin to win transfusion, both the donor and the recipient exhibit abnormalities of myocardial tissue deformation with ventricle-specific changes evident based on loading conditions. Donor LV systolic function is hyperdynamic due to hypovolemia and selective ejection into a low-resistance cerebrovascular circuit while the donor RV selectively ejects into a high-resistance placental circuit. Recipient RV and LV are both globally depressed with systolic and diastolic dysfunction [51].

### Tetralogy of Fallot

Assessment of the right ventricle in patients with Tetralogy of Fallot following surgical repair has shown a significant reduction in global longitudinal strain and strain rate vs. controls [52]. The reduction in global longitudinal strain rate was significantly lower in adult patients with Tetralogy when compared to children with Tetralogy. This is most likely due to reduced cardiac contractility with advancing age, whether this process is accelerated in the RV of patients with Tetralogy or not is unclear.

A recent study by van der Hulst *et al.* demonstrated the potential of 2DSTE to give new insight into the mechanism of RV dysfunction in tetralogy [53]. In this study they demonstrated that time to peak strain in the RV inlet remained normal whereas in the RV outlet there was a significant delay. The result of this was a significant decrease in the RV time delay with subsequent significant impairment in RV performance.

Longitudinal strain of the LV is reduced in adults following TOF repair suggesting subclinical myocardial damage of the LV. Abnormal torsion and strain pattern of the LV have also been observed in patients without symptoms of cardiac failure [54, 55]. In these studies LVEF was normal and not different from controls indicating the ability of STE to detect more subtle myocardial dysfunction.

### Fetal

The relatively low frame rates used for speckle tracking echocardiography remain the limiting factor for its use in assessment of fetal cardiac function. Data has however been produced for normal values for both longitudinal and circumferential strain in fetuses [56]. The majority of fetal studies use velocity vector imaging software. Germanakis *et al.* have recently published data for normal values of RV and LV longitudinal strain and LV:RV strain ratios. Fetuses with hypoplastic left heart had the lowest [0.29], and those with Ebstein the highest [1.55], LV:RV ratio. Additionally they found increased LV strain in aortic coarctation and aortic stenosis, but not in one developing important mitral regurgitation. Increased right ventricular loading in a fetus developing pulmonary regurgitation was associated with increasing RV strain [57]. Brooks *et al*. have looked at right ventricular function in fetal hypoplastic left heart syndrome. They found that the ratio of longitudinal to circumferential deformation was reduced in HLHS compared with the normal right ventricle and equivalent to the normal left ventricle. They conclude that the fetal right ventricle in HLHS becomes more spherical because of increased RV diameter. It has relatively reduced longitudinal compared with circumferential deformation and an increased reliance on atrial contraction for ventricular filling. These findings are similar to postnatal changes observed in the systemic right ventricle in palliated congenital heart disease, suggesting that ventricular remodelling is initiated in fetal life [58].

### Biventricular Pacing and Dyssynchrony

2DSTE has been used to optimise biventricular pacing. A case report recently published describes it use in implantation of a biventricular pacemaker in a nineteen-month-old child because of intractable heart failure. By analysing a 17-segment model using strain analysis of the left ventricle they were able to place the left ventricular lead at the latest activated segment [59]. Another report in Europace describes two children aged 7 weeks and 4 months, with transposition of the great arteries and ventricular septal defect, and double outlet right ventricle with pulmonary stenosis, who developed heart block after surgery [60]. Both developed heart failure with conventional pacing and speckle tracking echocardiography was used to synchronize and optimise pacing with resolution of heart failure.

### Applications Outside of Congenital Heart Disease

Although this review focuses on imaging in the malformed heart it would be amiss not to mention some of the other applications of 2DSTE.

A large amount of work has been done in using speckle tracking echocardiography to detect subclinical myocardial dysfunction that is not detected using traditional echocardiographic parameters. This has been demonstrated in patients with hypertrophic cardiomyopathy and other cardiomyopathies [61, 62] in patients following heart transplant and in patients receiving chemotherapy [63, 64]. A group from the Leuven cancer institute have looked at myocardial function using speckle tracking in children following foetal chemotherapy exposure, they did not find a significant difference in strain values in their patient group vs. normal controls [65].

Recently it has been used to detect myocardial dysfunction related to iron overload in patients with Beta-Thalassemia [66]. Basu *et al*. assessed 15 paediatric patients with septic shock admitted to the intensive care unit. They demonstrated that 2DSTE had the ability to detect a number of significantly impaired measures of ventricular performance in children with sepsis, not appreciated by conventional echocardiography. These children demonstrated significant differences in global longitudinal and circumferential strain and strain rate whilst demonstrating no significant difference in ejection fraction and fractional shortening [67].

2DSTE has demonstrated the ability to detect subclinical myocardial dysfunction in a number of conditions. This is an area where this new technology will play a significant role in the future in management of these patient groups.

2DSTE is also able to identify regional wall motion abnormalities, this has been largely used in adults with coronary artery disease but its use has been suggested in patients with Kawasakis disease and ALCAPA [68, 69]. 

### Tissue Motion Annular Displacement (TMAD) 

**Fig. (5):**
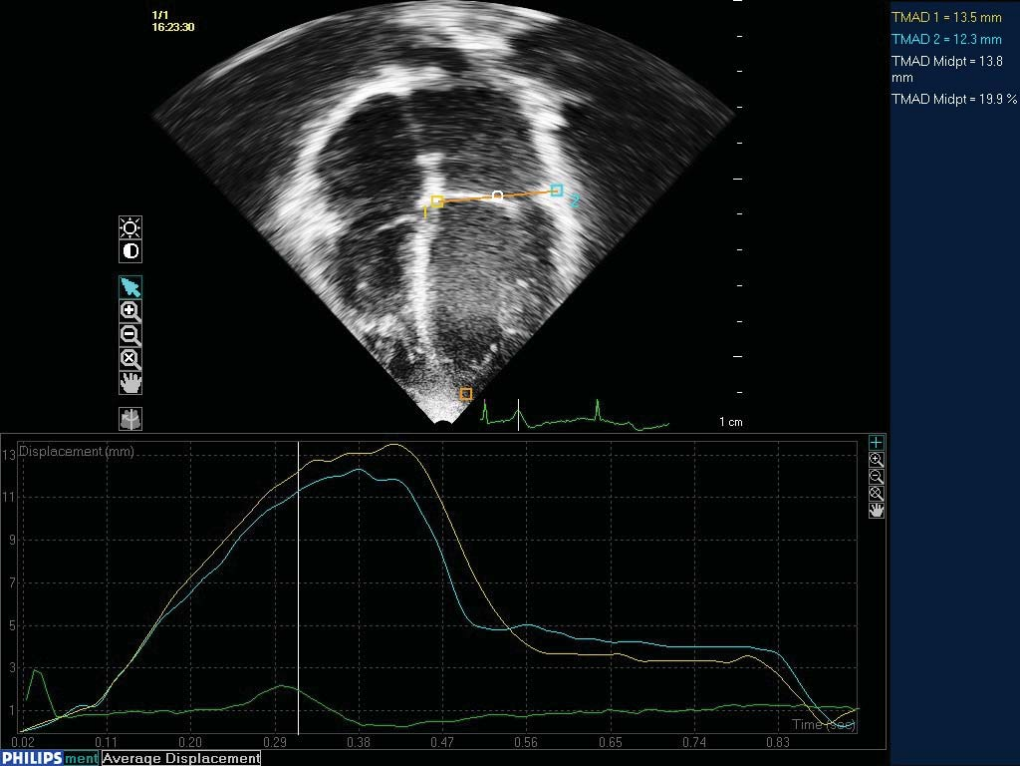
Four chamber apical view using two dimensional trans-thoracic echocardiography. Three points are manually selected at the medial mitral annulus [1], the lateral mitral annulus [2] and the apex [orange square]. Using speckle tracking technology these points are tracked through the cardiac cycle. This gives a measure of displacement [top right corner] and a graphic display of maximum displacement and time to peak contraction. The TMAD midpoint has show strong correlation with CMRI derived EF.

This is a simple and accurate method available through QLAB (Phillips) for assessing cardiac function in normal children. This uses 2DSTE to track the displacement of the mitral or tricuspid valve annulus through the cardiac cycle. During off line analysis of an apical four chamber view three points of interest are placed on the lateral aspect of the valve annulus, the medial aspect and the ventricular apex. The value obtained gives a measure of the displacement of the valve towards the ventricular apex. This value is similar to TAPSE but is obtained with 2DSTE and is angle independent. No data has been published using TMAD in congenital heart disease. Unpublished data from our institution has shown TMAD to be an easy, reproducible method for assessing LV function in normal children. TMAD of the mitral valve annulus correlated with MRI derived LVEF [r=0.69] and was superior to M-Mode derived EF [r=0.33]. This is a method that once validated will have a significant impact on bed side assessment of cardiac function (Fig. **5**).

### Three Dimensional Speckle Tracking Echocardiography

**Fig. (6):**
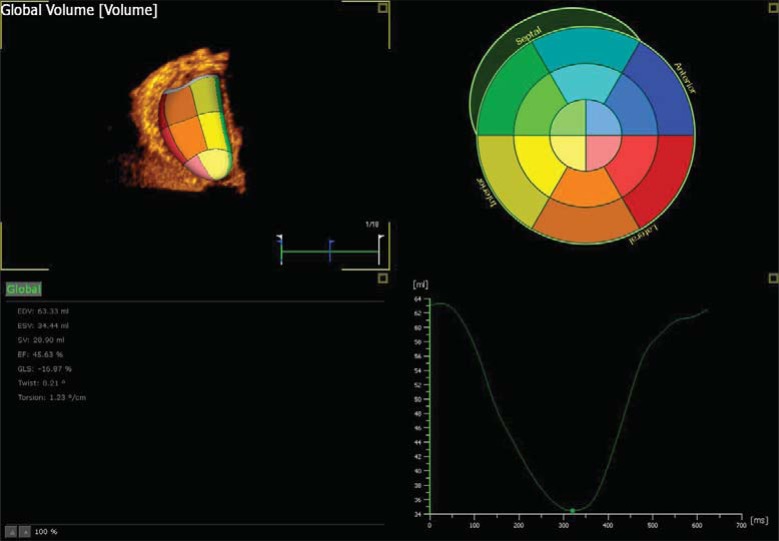
Three dimensional speckle tracking echocardiography of the left ventricle using Tomtec. Image shows three dimensional image of the left ventricle in the top left corner with a colour display of individual segments. Information automatically generated is displayed in the bottom left corner. EDV – end diastolic volume, ESV – End systolic volume, SV – stroke volume, EF – Ejection fraction, GLS – Global longitudinal strain, twist and torsion. A graphic display of change in volume over time is displayed in the bottom right corner. The software allows one to analyse various aspects of strain and displacement which are displayed graphically when selected.

Real time 3D has become available due to advances in transducer and beam forming technology. With current 3D technology there is a trade off for spatial and temporal resolution which will affect the data acquired. The benefit of 3D is simplicity of acquisition within one heart beat, it is useful for volumetric assessment, complex shapes and in arrhythmias. Future developments in 3D technology allowing acquisition with higher spatial and temporal resolution are needed. This is currently a research tool but with advancing technology holds promise for evaluation of ventricular function in malformed hearts (Fig. **6**).

Data has been published in which 3D speckle tracking has been used to quantify left ventricular volumes [70]. Nesser *et al*. have compared three-dimensional speckle tracking echocardiography with cardiac magnetic resonance and found that three-dimensional speckle tracking echocardiography measurements were in close agreement with the cardiac magnetic resonance reference values, in the presence of an adequate transthoracic two-dimensional acoustic window. To achieve automated assessment of left ventricular volumes and function with three-dimensional speckle tracking ultrasound, shortcomings have to be overcome such as low temporal resolution and random noise affecting the ability to track speckles during the cardiac cycle.

3D STE has also been used in assessing left ventricular mechanical dyssynchrony during CRT in heart failure patients [71].

## CONCLUSION

New developments and new technology will continue to emerge in the field of echocardiography. Strain analysis during stress echocardiography and three-dimensional imaging with shape analysis will advance our understanding of ventricular function in the malformed heart. It remains important, however, to have a good understanding of traditional methods of echocardiographic assessment of ventricular morphology and function. Although current developments hold exciting prospects for the future they are not yet at the stage where relevant clinical decisions can be made using them. When introducing new technology into your clinical environment it is important to be aware of the learning curve you will need to face. Image quality and validation of data obtained is essential before application in a clinical setting. Using this review and other similar articles to glean as much information as possible about image optimisation would be a good place to start. And, as stated previously, electronic media will play a large role in demonstrating the dynamic images produced using this technology.
